# Bromeliad Selection by Two Salamander Species in a Harsh Environment

**DOI:** 10.1371/journal.pone.0098474

**Published:** 2014-06-03

**Authors:** Gustavo Ruano-Fajardo, Sean M. Rovito, Richard J. Ladle

**Affiliations:** 1 Museo de Historia Natural, Escuela de Biología, Facultad de Ciencias Químicas y Farmacia, Universidad de San Carlos de Guatemala, Ciudad de Guatemala, Guatemala; 2 Museum of Vertebrate Zoology, University of California, Berkeley, California, United States of America; 3 Sector Diversidade Biológica e Conservação nos Trópicos, Instituto de Ciências Biológicas e da Saúde, Universidade Federal de Alagoas, Maceió, Alagoas, Brazil; 4 School of Geography and the Environment, Oxford University, Oxford, United Kingdom; University of Sao Paulo, Brazil

## Abstract

Bromeliad phytotelmata are frequently used by several Neotropical amphibian taxa, possibly due to their high humidity, microclimatic stability, and role as a refuge from predators. Indeed, the ability of phytotelmata to buffer against adverse environmental conditions may be instrumental in allowing some amphibian species to survive during periods of environmental change or to colonize sub-optimal habitats. Association between bromeliad traits and salamanders has not been studied at a fine scale, despite the intimate association of many salamander species with bromeliads. Here, we identify microhabitat characteristics of epiphytic bromeliads used by two species of the *Bolitoglossa morio* group (*B. morio* and *B. pacaya*) in forest disturbed by volcanic activity in Guatemala. Specifically, we measured multiple variables for bromeliads (height and position in tree, phytotelma water temperature and pH, canopy cover, phytotelma size, leaf size, and tree diameter at breast height), as well as salamander size. We employed a DNA barcoding approach to identify salamanders. We found that *B*. *morio* and *B. pacaya* occurred in microsympatry in bromeliads and that phytotelmata size and temperature of bromeliad microhabitat were the most important factors associated with the presence of salamanders. Moreover, phytotelmata with higher pH contained larger salamanders, suggesting that larger salamanders or aggregated individuals might modify pH. These results show that bromeliad selection is nonrandom with respect to microhabitat characteristics, and provide insight into the relationship between salamanders and this unique arboreal environment.

## Introduction

Bromeliads are epiphytic plants of the New World tropics, with the exception of one species that is found in Africa [1]. Their ecological importance is primarily associated with the possession of phytotelmata (singular phytotelm =  “plant that holds water”), which have the ability to collect water and nutrients [2]–[4]. This characteristic structure is utilized by a wide range of taxonomically diverse species, including bacteria, fungi, insects, amphibians, reptiles, and other plants [3], [5], [6], [7], [8]. Moreover, phytotelmata are considered as ‘biodiversity amplifiers’: their complex architecture and ability to damp environmental fluctuations can generate and harbor high levels of species diversity [9].

Amphibian species from several families are associated with forest bromeliads (e.g. [7], [10]). Some of these species utilize bromeliads over their entire life span (bromeligen species), while others (bromeliculous species) only use phytotelmata for reproduction or as a refuge from adverse environmental conditions [11]. Interaction with bromeliads have been particularly important in the evolution and ecology of several Neotropical amphibian groups (e.g. [7], [12], [13], [14]). Several bromeliad traits are associated with the presence of anurans, including humidity, water availability and phytotelmata size [15]–[18]. More generally, the high microhabitat stability of phytotelmata makes them potentially important refuges during adverse environmental conditions. For example, salamanders in cloud forests may use phytotelmata as refuges from extreme temperature fluctuations [19]. Nevertheless, microhabitat preferences of Neotropical salamanders are not well studied, leading Blankers *et al*. [10] and Wake [14] to suggest recently that more research is needed to identify fine scale associations, especially in areas where salamanders use bromeliads at high frequencies and that are threatened with habitat modification or are restricted to narrow elevational zones.

A good candidate organism for such fine-scale studies is the genus *Bolitoglossa*, the largest genus of tropical salamanders, distributed from northern Mexico to tropical South America [20] and containing more than 120 species [20], [21]. *Bolitoglossa morio* is a generalist that is associated with pine-oak forests and secondary habitats in the central highlands of Guatemala. In the west of its distribution, *B. morio* is found in a range of microhabitats, including the spaces under logs and rocks and, occasionally, in the phytotelmata of bromeliads [22]. Microhabitat use and species identity in the east of its range is much more uncertain, being complicated by possible co-existence with the newly described congeners *Bolitoglossa eremia, B. pacaya* and *B. kaqchikelorum* [23] from the range of what was formerly *B. morio*.

The objective of this study is to identify the characteristics of bromeliad phytotelmata that are used by salamanders of the *Bolitoglossa morio* complex in an active quaternary volcanic region of eastern Guatemala. Specifically, we evaluate: 1) whether differences exist in characteristics of bromeliads selected by two sympatric salamander species; 2) if the presence of salamanders is associated with larger phytotelmata, and; 3) whether bromeliad phytotelm characteristics affect the size of salamanders that they harbor.

## Materials and Methods

### Ethics Statement

Collected samples where made under the permit # 041/2008 and transport of tissue for DNA analysis under the permit # 011/58 issued by the Consejo Nacional de Áreas Protegidas (CONAP) of Guatemala. Samples were conducted according to relevant national and international guidelines. N/A Committee on the Ethics of animals experiment does not exist at Universidad San Carlos de Guatemala (USAC). Collection and euthanasia of salamanders where made under the permit #041/2008 (CONAP). All individuals were euthanized under MS-222 anesthesia, and all efforts were made to minimize suffering.

### Study site

**Figure 1:**
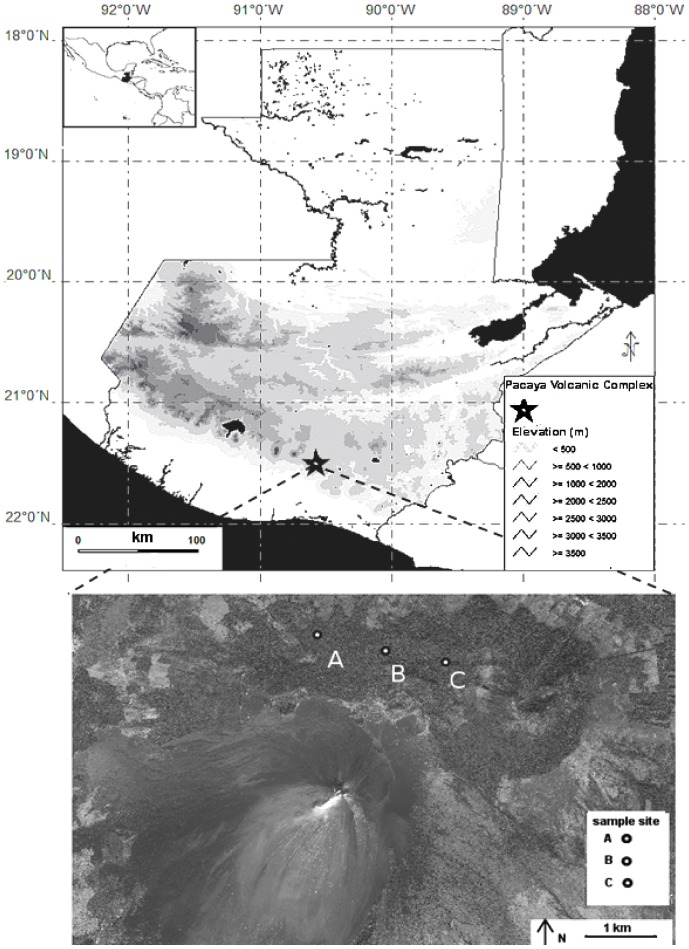
Map of the Pacaya volcanic complex and sampled localities.

Pacaya National Park (*El Parque Nacional Volcán de Pacaya* - PNVP) is located in the municipalities of Amatitlán and Villa Canales of the Department of Guatemala, and in San Vicente Pacaya, Department of Escuintla. It covers an area of approximately 1,800 hectares. The park is located in a temperate life zone considered as Subtropical Moist Forest [24]. The most widespread plant community in the area is broadleaf or broadleaf mixed forest, which includes species such as *Quercus oocarpa*, *Chiranthodendron pentadactylum* and *Annona sp*. (F. Castro unpublished data). Its main crater is active and reaches a height of 2,552 meters above sea level. Volcán Pacaya, which consists of a series of craters, cones and domes, is considered to be a volcanic complex. The sample area within the complex was on Cerro Chiquito (14°39′75.5″N, 90°60′37.2″W) and Cerro Grande (14°39′30.2″N, 90°59′61.7″W), between 2000 to 2336 m elevation (Fig. 1). This area is not currently volcanically active and its origin is linked to a gradual extrusion of lava.

### Study organisms

We recorded all species of *Bolitoglossa* in bromeliads with phytotelmata. We specifically focused on two species of bromeliads (Bromeliaceae), *Tillandsia guatemalensis* and *Werauhia werckleana*. Species in the *B. morio* group are regarded as generalists that are linked to pine-oak or broadleaf forests in the central highlands of Guatemala or to secondary habitats [22]. They occupy microhabitats created by logs, rocks and are occasionally found in bromeliads. However, in highly disturbed areas such as Cerro Grande on Volcán Pacaya (due to volcanic activity), bromeliads frequently host high-density salamander populations [19].

### Sampling

Fieldwork took place in the dry season from January to April of 2008. The replicability of the soil environmental conditions was defined by a pre-sampling to ascertain whether natural discontinuities exist between the temperature and humidity of the soil along the altitudinal gradient of the volcano. There were no significant difference for these variables (t-test, p = 0.05) indicating that sampling could be validly performed in any forested area within the sampling site. We used a path sampling method to look for associations between salamander species and particular microhabitat characteristics [25].

Individual bromeliads were used as sampling units. In total, 45 bromeliads were sampled across three sites (15 bromeliads per site A, B and C) using a saw. Sampling was always performed between 10:00–16:00 h; *Bolitoglossa* are nocturnal and are confined to refuges during the daytime. We took environmental data from all bromeliads, including those which did not contain salamanders. We restricted sampling to bromeliads ≤7 m above the ground. We recorded data on bromeliad distribution on the tree (height and location on the tree and number of bromeliads), tree diameter at breast high (DBH), species of bromeliad, tank diameter (tank size), size along the second leaf of the bromeliad, canopy coverage, relative humidity (HR), microhabitat temperature within the center of the bromeliad water [T_m_] and ambient air temperature [T_A_] were measured with a “VWR Humidity/Temperature Digital Thermometer”, and pH of water inside the bromeliad was measured with a Hanna Instruments pH-meter Checker. We also recorded geographic coordinates, altitude, soil pH, and soil temperature at each tree. Finally, we searched for salamanders by shaking the bromeliad into a white plastic container. Salamanders were euthanized following standard procedures, and liver tissue samples were collected in 95% ethanol for molecular analyses. Finally, each specimen was preserved in 10% formalin for 24 hours and stored in 70% ethanol. Liver tissue samples and voucher specimens were deposited in the Natural History Museum (MUSHNAT) of San Carlos University, Guatemala (USAC). We measured distance from snout to posterior end of vent (SVL) of each preserved salamander with dial calipers.

### Molecular Analyses

Campbell *et al*. [23] described several new species of salamanders of the *Bolitoglossa morio* group from southeastern Guatemala, including *Bolitoglossa pacaya* from Volcán Pacaya. Preliminary molecular analyses showed the presence of two different mtDNA haplotypes on Volcán Pacaya, indicating the likely presence of different species. Given that many of the specimens that we collected were not adults, we used a molecular barcoding approach to allocate specimens to named species. Molecular lab work was done in the Evolutionary Genetics Lab, Museum of Vertebrate Zoology (MVZ), University of California, Berkeley. We extracted DNA using a guanidine-thiocyanate protocol (available upon request). We sequenced the large subunit ribosomal rRNA gene (16S, 508 bp) for 57 specimens and the cytochrome b gene (cyt b, 803 bp) for 6 specimens by using primers MVZ117 and MVZ98 for 16S [26] and primers MVZ15 and MVZ16 for cyt b [27]. Reactions were run at 94°C for 2 min, 38 cycles of 94 °C for 30 sec, 48 °C for 30 sec (16S) or 1 min (cyt b), and 72 °C for 1 min, with a final extension at 72 °C for 8 min. All sequences were deposited in GenBank (Table S1). We aligned the cytb sequences with available sequences for other populations formerly assigned to *B. morio* from GenBank using the program MUSCLE 3.6 [28] and assigned the haplotypes found in our dataset to named species based on similarity to GenBank sequences. We then compared 16S haplotypes between these samples for which we determined species identification to all other 16S haplotypes in order to assign a species name to all individuals sampled for which tissue was available. In the phylogenetic analyses of Campbell *et al*. [23], *Bolitoglossa morio* and the newly described *B. kaqchikelorum* are not reciprocally monophyletic. The two species are similar in external morphology, calling into question the taxonomic status of *B. kaqchikelorum*. Multiple individuals from Volcán Pacaya were most similar to sequences of these two species. We choose to apply the name *B. morio* to the individuals from Volcán Pacaya until the taxonomic status of *B. kaqchikelorum* is resolved through further analyses.

### Data analyses

We performed Principal Component Analysis (PCA) to identify which variables are associated with the presence of salamanders. The Kaiser–Guttman criterion was used to determine how many axis were worth analyzing based on computing the mean of all eigenvalues and interpreting axes with eigenvalues larger than the mean. We also used Pearson's correlation coefficient to choose between the variables that were associated with each axes (r>0.5; p<0.05). Finally, we analyzed the components that explained the most variance and descriptively related the presence of different species of salamanders.

We used logistic regression to examine the effect of 7 independent variables (listed above) on the probability of presence of salamanders in 40 sampled bromeliads for each species and 45 bromeliads for all salamanders, regardless of species. Highly correlated [r>0.7] variables were excluded. One goal was to determine which factors, alone and in combination, had significant effects on the probability of the presence of each species or on the probability of presence of salamanders, regardless of species. A second goal was to develop a model with which to predict the species likely to be encountered. Specifically, we aimed to identify the variables that best predict the presence of each species as well as the presence of salamanders, regardless of species identity. The multiple logistic regression takes into account only the variables with significant effects to explain the variance in the dependent variable (salamander presence). Significance for each variable was evaluated with the Wald X^2^ statistic and model adequacy for the logistic regression models was assessed with the Hosmer-Lemeshow (HL) goodness of fit test [29].

We used a stepwise multiple linear regression to determine whether the body size of individuals (SVL) of *B. morio* was associated with bromeliad variables. Only variables that were significant when added to the model were retained (p<0.05). We used only the size of biggest salamander found in each bromeliad to avoid pseudoreplication. *Bolitoglossa pacaya* was excluded from this analysis because only three adults were found. We evaluated the significance of the regression model using an ANOVA. All analyses were performed using the R Environment for Statistical Computing [30]. In addition, we used Kruskal-Wallis (KW) nonparametric tests for *B. morio* SVL and encounter rate to compare salamander size and abundance between sample sites. Dunn's HSD nonparametric tests and Mann-Whitney U test were used to compare environmental variables between sample sites.

## Results

### Species identification

Molecular analyses of 57 individuals show that *Bolitoglossa morio* (n = 46) and *B. pacaya* (n = 11) co-exist in bromeliads in all the PNVP area. We found both species (*B. morio* n = 31, *B. pacaya* n = 9) at all three sampling sites as well (Table 1). Both salamander species were commonly found in the same bromeliad; in fact, 6 of 7 bromeliads harboring *B. pacaya* contained both species. More generally, salamanders commonly appear to aggregate, with 8 of 22 bromeliads containing between 2 to 8 individuals. The encounter rate of salamanders in bromeliads on the Volcán Pacaya was 0.37 salamanders/bromeliad (+0.07 s.d.). The encounter rate of salamanders in bromeliads did not differ significantly across the three sampling sites (Kruskal-Wallis test, KW = 1.217, p = 0.5441).

**Table 1:** Number of individuals and presence encounter rates during bromeliad sampling at three sample sites.

Salamander species	Site A	Site B	Site C	Total	ER (s.d.)
*Bolitoglossa morio*	18	7	6	31	0.35 (±0.10)
*Bolitoglossa pacaya*	1	4	4	11	0.24 (±0.13)
All salamanders^a^	19	22	13	54	0.37 (±0.07)
**Bromeliad species**					
*Werauhia werckleana*	15	12	1	28	0.24 (±0.23)
*Tillandsia guatemalensis*	0	3	14	17	0.37 (±0.34)

Encounter rate (ER), measured as proportion of bromeliads containing salamanders, by salamander species and bromeliad species. All data of presence given as mean ± standard deviation (s.d.) and based on three replicates patches of forest (n = 15).

a Including salamanders with no molecular taxonomic identification.

### Microhabitat Selection

Six continuous variables (height above ground, ambient temperature [T_A_], microhabitat temperature [T_M_], pH, width and length of the bromeliad), were used for PCA. Principal component axes one (PC1) and two (PC2) had explanatory variances over 20% and were supported by the Kaiser–Guttman criterion.

**Figure 2:**
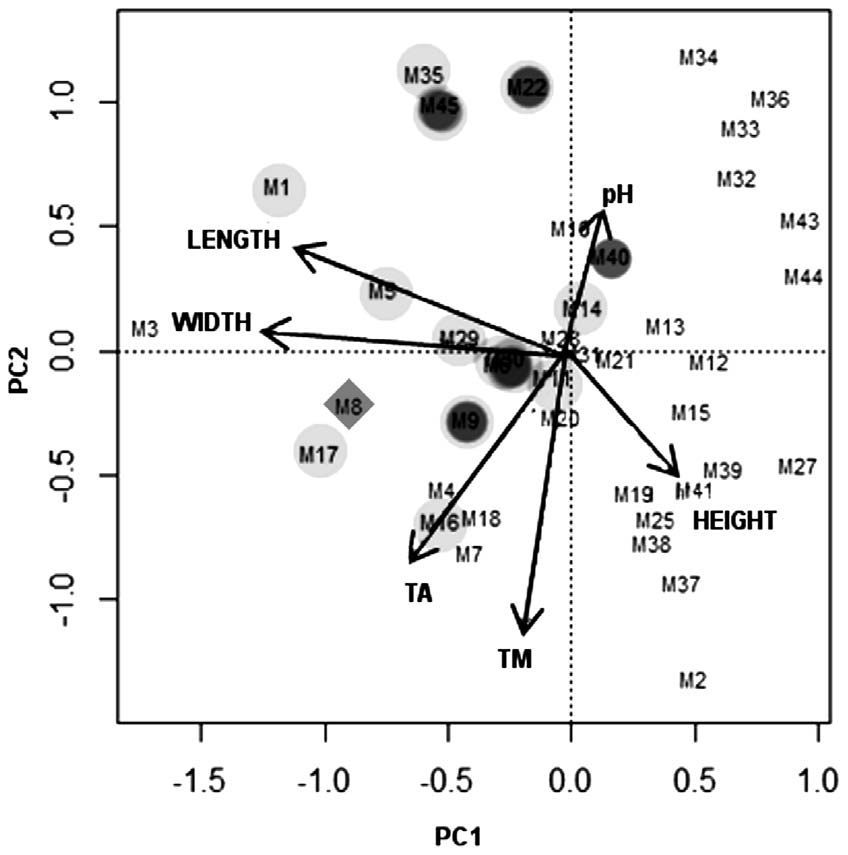
Biplot of PC2 vs PC1 from variables describing bromeliads. Grey circle = *B. morio*, black circle = *B pacaya* and black diamond = unidentified (juveniles with no tissue available). Height above ground = Height, ambient temperature = T_A_, microhabitat temperature = T_M_, Tank size diameter = width, size along the second leaf of the bromeliad = Length.

PC1 is negatively correlated with bromeliad length (r = −0.85) and width (r = −0.91) (Fig. 2) and PC2 is negatively correlated with microhabitat temperature (r = −0.80). Examination of characteristics of bromeliads containing salamanders shows that three variables might be related to the presence of salamanders (Table 2). Salamanders were found more often in bromeliads with larger tank size (diameter) and leaves (lower values of PC1) and in bromeliads with lower temperature (higher values of PC2). Mean values for the bromeliads used by salamanders were 33.1 cm tank size (95% CI, 26.45 cm–39.84 cm) and 16.9 °C bromeliad temperature (95% CI, 15.89°C–17.45°C). These parameters were consistent with the results of the PCA.

**Table 2:** Measurements of microclimatic conditions and phytotelm characteristics in bromeliads with and without salamanders.

Salamander	Absence		Presence			
Variable	Mean (s.d.)	n	Mean (s.d.)	n	Test	P
Width (cm)	20.3 (±14.8)	23	33.5 (±14.4)	22	W = 94.5	0.0003*
Length (cm)	32.1 (±16.7)	23	55.4 (±22.0)	22	W = 94.5	0.0003*
pH	5.7 (±0.6)	23	5.8 (±0.6)	22	t = −0.5245	0.6
Tm (°C)	17.6 (±1.5)	23	16.7 (±1.7)	22	t = 1.8918	0.06^.^
Ta (°C)	19.6 (±2.4)	23	19.9 (±1.9)	22	t = −0.4419	0.6
HR (%)	68.2 (±14.3)	23	66.6 (±12.6)	22	t = 0.3949	0.7
DBH (cm)	27.5 (±12.6)	23	32.8 (±30.9)	22	W = 266.5	0.57
Height (cm)	358.4 (±160.7)	23	318.1 (±160.1)	22	W = 301.5	0.3
Canopy coverage (%)	80.9 (±6.2)	23	78.8 (±8.7)	22	W = 278.5	0.57

Tank diameter  =  Width, size along the second leaf of the bromeliad  =  Length, microhabitat temperature  = T_m_, ambient air temperature  =  T_A_, Relative humidity  =  HR, tree diameter at breast high  =  DBH, Height of the bromeliad in tree  =  Height.

*Significance level for (W) Mann-Whitney pairwise comparisons or t-test (p<0.05).

Significance level (p<0.1). All data given as mean ± standard deviation (s.d).

In the logistic regression analysis, relationships of salamander presence with phytotelm size and bromeliad temperature were selected by stepwise AIC for each species and for overall salamander presence (Table 3). For both *Bolitoglossa morio* and all salamanders together, phytotelma size was significantly associated with salamander presence (Table 3). *Bolitoglossa pacaya* and all salamanders together showed bromeliad temperature as significantly associated with salamander presence.

**Table 3:** Multiple logistic regressions for the presence of salamanders in bromeliads selected by stepwise by AIC. Variables significant at the α = 0.05 level are indicated with an asterisk (*).

Species	Variable	Estimate	Std. Error	Wald z-statistic	Pr(>|z|)
All Salamanders	(Intercept)	13.44	7.394	2.028	0.042 *
	Width	0.092	0.036	2.553	0.011 *
	Bromeliad T°C	−0.54	0.249	−2.167	0.030 *
	Canopy coverage	−0.092	0.053	−1.537	0.1243
*Bolitoglossa pacaya*	(Intercept)	17.11368	9.41918	1.817	0.0692
	Width	0.04502	0.0282	1.596	0.1104
	Bromeliad T°C	−0.75703	0.38199	−1.982	0.0475*
	Canopy coverage	−0.10022	0.07295	−1.374	0.1695
*Bolitoglossa morio*	(Intercept)	2.857	3.724	0.767	0.443
	Width	0.0778	0.033	2.324	0.020 *
	Bromeliad T°C	−0.308	0.223	−1.378	0.168

Tank diameter  =  Width, bromeliad temperature  = T_m_.

*Significance level (P<0.05) for Wald z-statistic test.

**Figure 3:**
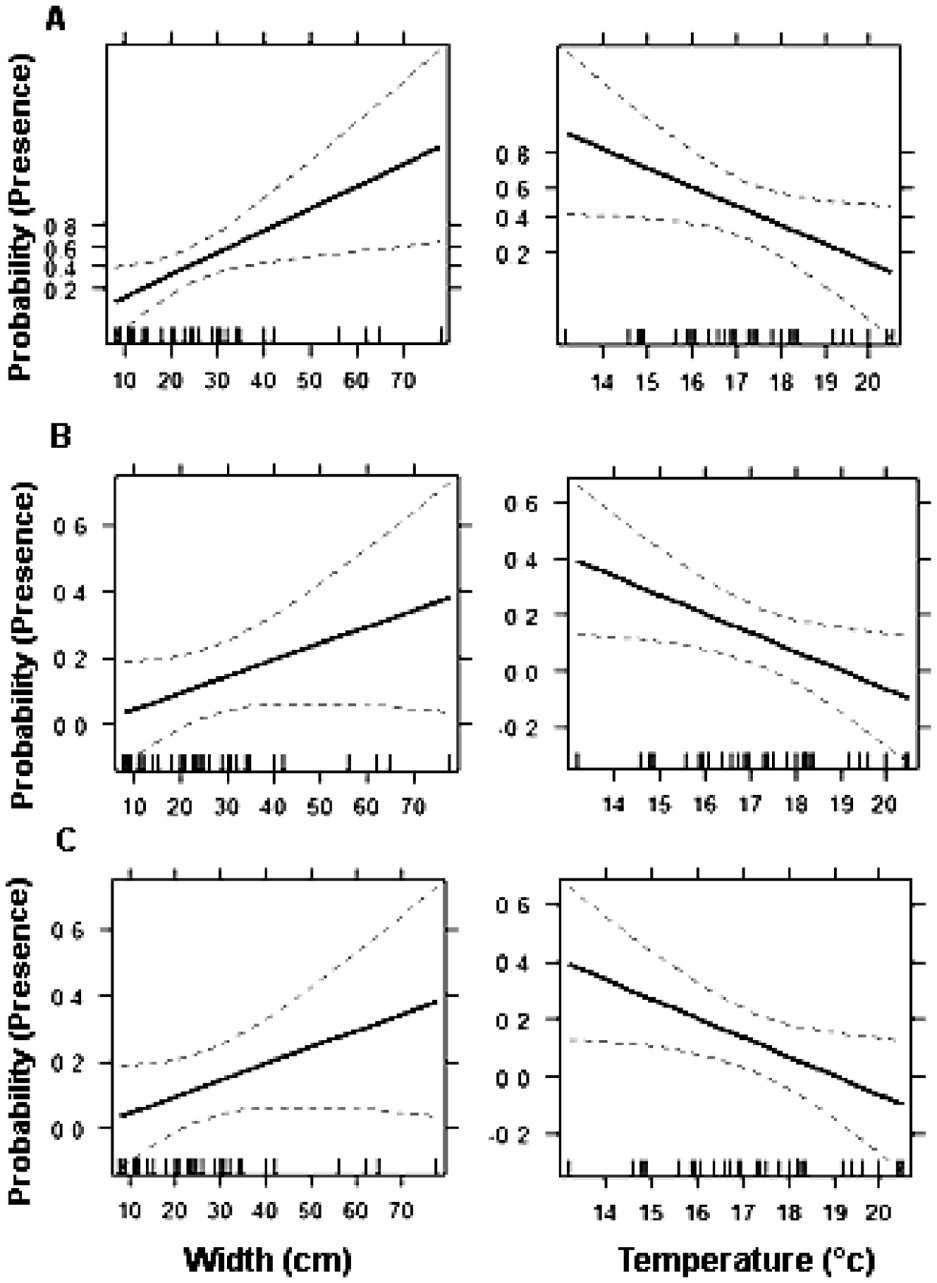
Interaction of bromeliad width (cm) and temperature (°C) in the logit model fit to presence data. The vertical axis is labelled on the probability scale, and a 95-percent pointwise confidence interval is drawn around the estimated effect. A) All salamanders B) *B. morio* and C) *B. pacaya*.

The Hosmer-Lemeshow (HL) goodness of fit test was not significant (p>0.05) for each model, showing that each model provides a good fit to explain salamander presence (all salamanders: **χ^2^** = 10.84, DF = 8, p = 0.26; *B. morio*: **χ^2^** = 11.68, DF = 8, p = 0.16; *B. pacaya*: **χ^2^** = 4.49, DF = 8, p = 0.809). We plot the effects displayed by interactions of the variables for each model and present the probability of presence in bromeliad by overall salamanders and *B. morio* (Fig. 3).

**Figure 4:**
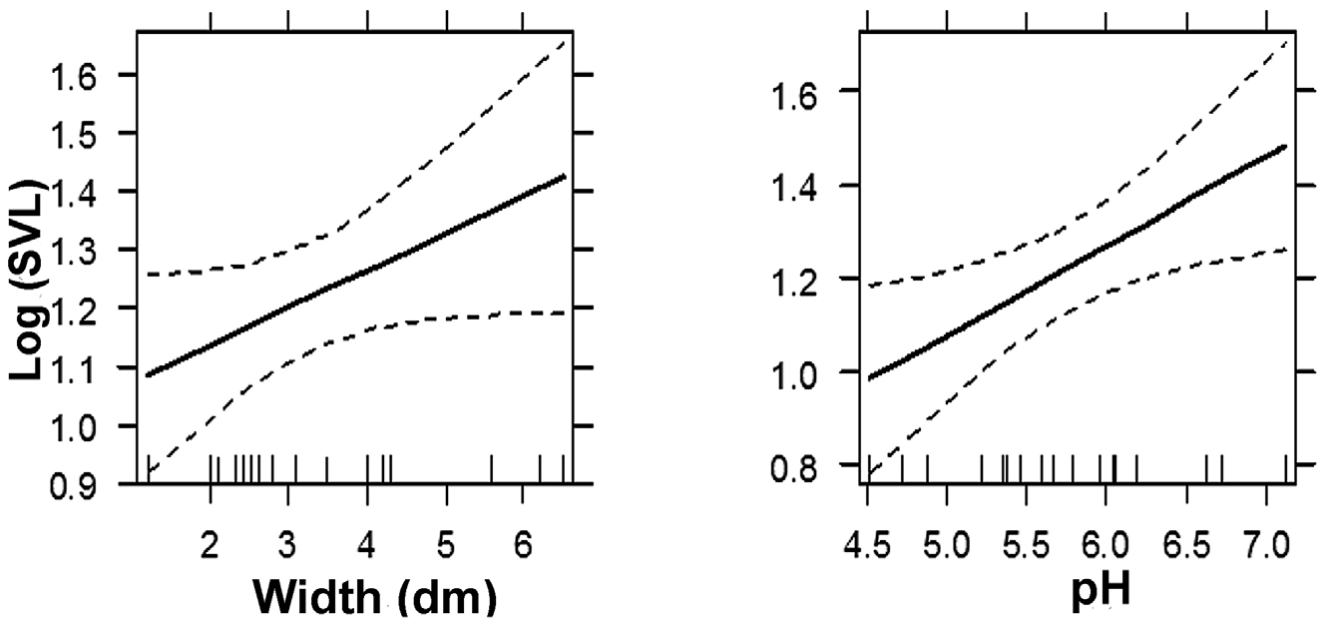
Interaction of bromeliads variables in the model fit to the SVL (mm) of *B. morio*. The horizontal axis is labeled as bromeliad temperature (°C) and pH. Ninety-five percent confidence intervals are draw around the estimated effect.

Variables that influenced the size of *Bolitoglossa morio* in bromeliads included pH and bromeliad width (Table 4). The adjusted correlation (R^2^ = 0.2824) indicates that the model explains a fair amount of the variance of *B. morio* size. The ANOVA for the model indicates a significant fit to the data (F = 4.542; DF = 16; p = 0.027). We also plot the effects display for the interactions of SVL and the variables for the model (Fig. 4). The model suggests that bigger bromeliads and those with higher pH harbor bigger salamanders.

**Table 4:** Multiple regression for SVL of *Bolitoglossa morio* salamanders in bromeliads.

Variable	Estimate	Std. Error	T	Pr(>|z|)
(Intercept)	−2.2294	1.9133	−1.165	0.261
Width	0.2852	0.127	2.249	0.039*
pH	0.7316	0.266	2.794	0.014*

Variables significant at the α = 0.05 level are indicated with an asterisk (*).

The size of *Bolitoglossa morio* was different for sampling sites (Table 5), specifically site C compared to site A (Dunn test, D = 10.61, p<0.05). Only juveniles of *B. morio* were found at site C. In addition, other variables were different between the sample sites (Table 5). These variables seem to be related with major scale conditions. For example, relative humidity is significantly higher at site C (Dunn test, D = 14.63, p = 0.01). This variable also appears to be important in the distribution of bromeliads, and may be responsible for higher number of sample of *Tillandsia guatemalensis* (Table 1) at site C (14) compared to site A (0) and site B (3). Furthermore, tank diameter of *W. werckleana* is larger than in *T*. *guatemalensis* (Mann-Whitney U test, z = 376, P = 0.002). A significative correlation between tank size and T_M_ was found for *T*. *guatemalensis* (Table 6), but not for *W. werckleana* (Table 7).

**Table 5:** Variables of bromeliads from three sampling sites.

Variables	Site A	Site B	Site C	KW Test	P
Width (cm)	33.9 (±18.2)	28.8 (±14.9)	17.6 (±9.5)	10.28	0.005*
Length (cm)	44.9 (±23.9)	49.8 (±19.3)	35.9 (±23.5)	4.81	0.09
pH	5.78 (±0.81)	5.78 (±0.57)	5.78 (±0.46)	26.1	0.87
Tm (°C)	17.6 (±1.19)	17.3 (±1.89)	16.5 (±1.72)	5.17	0.07
Ta (°C)	20.7 (±2.01)	20.5 (±0.87)	18.0 (±2.31)	10.17	0.006*
HR (%)	63.5 (±7.96)	58.5 (±13.02)	80.0 (±7.30)	22.57	0.00001*
DBH (cm)	21.7 (±11.07)	39.7 (±35.45)	28.9 (±12.0)	3.91	0.14
Height (cm)	300.87 (±158.4)	434.3 (±188.2)	280.9 (±70.23)	6.19	0.04*
Canopy coverage (%)	79.7 (±6.17)	82.12 (±7.39)	77.9 (±8.63)	2.2	0.33
*B. morio* (SVL)	3.26 (±0.77)	3.39 (±0.77)	2.71 (±0.18)	7.11	0.02*

*Significance level (P<0.05) for Kruskal-Wallis (KW) test.

**Table 6:** Spearman correlations between *T. guatemalensis* microclimatic conditions and bromeliad characteristics.

	Height	Width	Azi	Cover	DHC	HR	Length	Masl	pH	Ta	Tm
**Height**	1										
**Width**	0	1									
**Azi**	−0.3	−0.1	1								
**Cover**	−0.1	0.1	−0.1	1							
**DBH**	0.2	−0.1	0.1	0.2	1						
**HR**	−0.4	−0.1	0.1	0.3	−0.1	1					
**Length**	−0.1	0.8*	−0.4	0.1	−0.2	−0.2	1				
**Masl**	0	−0.3	0.3	−0.3	−0.1	0.1	−0.3	1			
**pH**	0	0	−0.3	0.3	−0.4	0	0	−0.3	1		
**Ta**	0.5	−0.1	−0.3	−0.3	0	−0.8*	0	−0.1	0.1	1	
**Tm**	0.2	−0.5*	−0.4	−0.1	−0.2	0.1	−0.4	0.2	−0.1	0.2	1

*p values with significance level of (p<0.05).

**Table 7:** Spearman correlations between *Werauhia werckleana* microclimatic conditions and bromeliad characteristics.

	Height	Width	Azi	Cover	DBBH	HR	Length	Masl	pH	Ta	Tm
**Height**	1										
**Width**	−0.3	1									
**Azi**	0.3	−0.4*	1								
**Cover**	0.3	−0.1	0.1	1							
**DBH**	0.2	0.1	−0.1	0.4*	1						
**HR**	0.2	−0.2	−0.1	−0.1	0.2	1					
**Length**	−0.1	0.6*	−0.2	0.3	0.2	−0.1	1				
**Masl**	0.3	−0.1	−0.2	0.3	0.3	0	0.2	1			
**pH**	0	−0.2	−0.1	0.1	0.1	0	0.1	0.3	1		
**Ta**	−0.2	0.2	−0.2	0.4	0	−0.6*	0.3	0	0.2	1	
**Tm**	−0.3	0.1	−0.4	−0.4	−0.2	−0.2	−0.2	0	−0.3	0.1	1

Height of the bromeliad in tree  =  Height, tank diameter  =  Width, disposition in the tree  =  Azi, Canopy coverage  =  Cover, tree diameter at breast high  =  DBH, Relative humidity  =  HR, size along the second leaf of the bromeliad  =  Length, meters above sea level  =  Masl, microhabitat temperature  = T_m_, ambient air temperature  =  T_A_.

*p values with significance level of (p<0.05).

## Discussion

### Species identification

Our results indicate that *Bolitoglossa* are found at high density in bromeliads on Volcán Pacaya. DNA barcoding indicated that two species are sympatric at all three sampling sites, with a higher proportion of *B. morio* individuals across sites in comparison with *B. pacaya*. These closely related species frequently shared the same bromeliad phytotelm and both species appear to select bromeliads with similar characteristics; multiple regression models for each species and for the entire dataset selected the same variables, suggesting that our results may be generalizable to other arboreal salamander species, or at least to other arboreal *Bolitoglossa* in Mesoamerica.

### Microhabitat Selection

Salamanders did not select bromeliads randomly with respect to phytotelm characteristics. More salamanders were found in bromeliads with larger phytotelmata and cooler water. This is broadly in concordance with previous studies that have reported that humidity, water availability, and phytotelm size have been reported to play a major role in anuran microhabitat selection [15]–[18]. Such correlations are potentially driven by the role of bromeliad phytotelmata as refuges from temperature extremes and low humidity [7], [12], [19]. Larger bromeliads species may retain water more effectively, maintaining higher humidity, buffering against external fluctuations in air temperature and having less variable thermal conditions [31]. Thus, ability to select larger bromeliads with adequate moisture may be especially important for salamander survival in extreme conditions, such as those that occur during the dry season (approximately December to May on Volcán Pacaya). Alternately, larger bromeliads may simply provide a larger volume of suitable microhabitat space for salamanders, independent of their role in buffering temperature and humidity fluctuations. Bromeliad size could also be correlated with other factors besides temperature that affect salamander presence, such as prey abundance, diversity of prey, or protection from predators. Experiments would be needed to establish causal relationships between tank size, temperature, and salamander presence.

For *Bolitoglossa morio and B. pacaya*, temperature and bromeliad size may both be important as a buffer to external conditions Indeed, studies suggest that bromeliads in a Guatemalan cloud forest provide cooler, less variable microclimatic temperatures compared to their surroundings, but that thermal variability within individual bromeliads is low [31]. Feder [31] observed no difference in temperature between bromeliads harboring two species of Guatemalan salamanders (*B. franklini* and *Dendrotriton bromeliacius*) and those without salamanders, and interpreted this to mean that bromeliads did not offer sufficient thermal diversity for effective thermoregulation. Although bromeliads were sampled at random, tank size was not reported. Furthermore, bromeliad size could be less important for salamander in a region of high water availability as a cloud forest.

Water relations are thought to limit thermoregulation in most species of salamanders because hydric requirements restrict salamanders to moist microhabitats such as bromeliads, which exhibit little temperature variation in cloud forest [31]. The presence of *Bolitoglossa morio* and *B. pacaya* was associated with a relatively wide range of bromeliad temperature (17.6+1.7 SD °C). This may have allowed sufficient thermal diversity in bromeliads for behavioral thermoregulation. Several species of Neotropical salamanders have been shown to select a narrow range of substrate temperature in thermal gradient experiments [31], demonstrating their capacity for thermoregulation when sufficient thermal diversity is available. Enhanced microhabitat stability in bigger bromeliads may thus be critical in sites such as Volcán Pacaya, where volcanic activity can cause severe environmental disturbance [19] and where the forest has less precipitation than cloud forest [24]. Nevertheless, it should be noted that other factors (not measured in this study) may also be associated with bromeliad size or may be independently important for salamander preference.

Campbell *et al*. [23] described *Bolitoglossa pacaya* as being significantly smaller than *B. morio*. The two species in our study appeared to be largely similar based on external morphology (Mann-Whitney test, U = 9.0; P = 0.63), although our sample size for *B. pacaya* was very small. If there is a major difference in body size between the two species, it was not reflected in their microhabitat preferences in our sample on Volcán Pacaya. Moreover, Campbell *et al*. [23] did not report any instances of sympatry between their newly described species of what was formerly *B. morio*, and the sympatry of two species on Volcán Pacaya raises the possibility that their series of *B. pacaya* may contain more than a single species. The presence of two morphologically similar sister species in microsympatry is unusual, because one species would be expected to be competitively superior and exclude the other [32]. The lack of separation by bromeliad species or by some variable related to bromeliad characteristics suggests that more subtle morphological or natural history differences between *B. morio* and *B. pacaya* that allow them to coexist. A similar situation has been found in other groups of tropical bolitoglossines, such as the genus *Thorius*, which may finely partition niche space through small but important differences in morphology between sympatric species [33]. Salamander size and morphology may be related to dietary overlap and the size of prey captured, as has been demonstrated for two species of temperate plethodontid salamanders [34]. A more detail morphological examination of *B. morio* and *B. pacaya* would be desirable to assess whether trophic partitioning is occurring. The large difference in encounter rate between the two species in our study suggests that they may have different natural histories, despite selecting bromeliads with similar characteristics.

The highest concentration of salamanders (>3 salamanders per bromeliad) occurred in bromeliads that were <3.8 m above the ground across sample sites. At a site in San Marcos, Guatemala, *Bolitoglossa morio* is found primarily in terrestrial habitats or under the bark of or inside standing trees and stumps, rather than in arboreal bromeliads [22]. The observed preference for bromeliads closer to the ground may reflect that *B. morio* is, in general, not a highly arboreal species and may use bromeliads extensively only when terrestrial conditions are not suitable, such as those on Volcán Pacaya. Furthermore, aggregations of more than three salamanders were commonly found in bromeliads in past surveys of Guatemala cloud forest [19].

Salamander morphology and microhabitat use have shown to be largely unrelated across plethodontids at a broad scale, including temperate and tropical subgroups. The Neotropical salamanders were an exception to this pattern, showing a significant association between morphology and microhabitat use, but no within species variation was taken into account [10]. In our study, larger *B. morio* were found in larger bromeliads with higher pH tank water. Individual and population level differences in microhabitat use have been reported in other salamander species. For example, the adults of the Red-spotted Newt (*Notophthalmus viridescens*) showed that aquatic adults from ponds varying in pH exhibited differences in pH tolerance and water preference [35]. Males of the bromeligen frog *Scinax perpusillus* in the south Atlantic Forest of Brazil showed a preference for bromeliads with higher pH (5.0–5.5 pH) [15]. The mean pH in bromeliads containing bigger *B. morio* was similar to those higher pH reports (5.8+0.6 s.d.).

Rain water typically has a pH of approximately 5.6, but gases from volcanic activity can lower the pH to as low as <4 in the Pacaya National Park (B. Oliva unpublished data). A laboratory study on an aquatic adult of the *Notophthalmus viridescens* showed that the effect of prolonged exposure to low pH (3) led to an inability to maintain sodium balance in an aquatic setting, causing loss of bodily sodium [36]. In addition, pH in stressful environments might affect locomotion of the stream-side salamander *Desmognathus ochrophaeus* [37]. Although salamanders probably do not spend time directly in tank water, they may be near to or in contact with water between bromeliad leaves much of the time. This suggests that larger salamanders may be selecting bromeliads with higher pH as an osmoregulatory strategy. It is possible that salamander presence or/and nitrogenous excretion modifies the pH of the bromeliad tank water, especially in cases of aggregation of multiple salamanders or in bromeliads with larger salamanders. Woodley *et al*. [37] showed that the presence of salamander in vitro humidity environment affected the pH in their petri dish and Romero *et al.* [38] point out that the bromeliculous frog *Scinax hayii* contributes nitrogenous waste to the bromeliad tank system through excretion. Given that our study is correlative in nature, we cannot distinguish between selection by salamanders for bromeliads with higher pH, selection for some other factor correlated with higher pH, or an increase in pH caused by salamander presence.

Bromeliad species identity does not appear to be important for the presence of salamanders; both *Bolitoglossa morio* and *B. pacaya* were found in both species of bromeliads present in the study area. However, the species of bromeliad may be important in determining the characteristics of salamanders found in arboreal microhabitats. We found smaller salamanders (only juveniles) at site C (Fig. 1), which also has higher relative humidity and lower ambient temperature (Table 5). The difference between numbers of *Werauhia werckleana* and *Tillandsia guatemalensis* sampled at the three sites seems to be related to this temperature and humidity difference (Table 1); relative humidity was higher at site C and *T. guatemalensis* was more abundant at this site (14 of 15 bromeliads sampled). These differences in environmental variables and relative abundance of bromeliad species between sites could be related to altitude, as site C was 200 m higher in elevation.

The presence of only juvenile salamanders in bromeliads at site C does not mean that larger individuals are not present at the site. If the lack of adults in bromeliads in our sample is not the result of small sample size alone, thermal availability of other microhabitats could explain the lack of adults in bromeliads. Large-scale environmental factors (ambient temperature and humidity) could control the distribution of bromeliads in the forest on Volcán Pacaya and also could make other microhabitats (e.g. under stones or logs) more favorable for adult salamanders in terms of temperature and humidity.

In summary, our results show that the presence of salamanders in bromeliads is nonrandom with respect to bromeliad characteristics and position, and that the correlations between bromeliad characteristics and salamander presence do not differ across two closely related species. The preferential use of cooler bromeliads may indicate that salamanders use bromeliad selection as a method of thermoregulation, or may simply be the consequence of selecting larger, more buffered bromeliads during the dry season. Salamanders could select bromeliads with higher pH, or bromeliad pH could be affected by salamander presence. Both bromeliad temperature and pH could either directly used to select bromeliads by salamanders, or could be correlated with other factors that salamanders use to select bromeliads. Differences in size of salamanders found in bromeliads may be related to both the environmental characteristics at a site, the relative abundance of different bromeliad species at the site, or both of these factors as well as their interaction. Our results show that bromeliad microhabitat characteristics may play an important role in structuring the composition of arboreal salamander communities. More research on the characteristics of bromeliads selected by other species of Neotropical plethodontids is needed to fully understand the role of phytolemata in buffering species from environmental extremes and shaping arboreal salamander communities across Mesoamerica.

## Supporting Information

Table S1 Voucher information and GenBank accession numbers for specimens of *Bolitoglossa* used phylogenetic analysis.
(PDF)
