# Supplementary material for: Bromeliad Selection by Two Salamander Species in a Harsh Environment
Source: PLoS One. 2014 Jun 3;9(6):e98474. doi: 10.1371/journal.pone.0098474 (PMC4043640; doi:10.1371/journal.pone.0098474)
Supplement: Table S1 — Voucher information and GenBank accession numbers for specimens of Bolitoglossa used phylogenetic analysis. (PDF) [file pone.0098474.s001.pdf]

Table S1. Voucher information and GenBank accession numbers for specimens of *Bolitoglossa* used in phylogenetic analysis.

Species	Locality	Voucher #	Field ID	Genebank 16S	Genebank Cyt-b
Bolitoglossa morio	Guatemala: Esquintla	USAC 1592	GRF014	KJ787694	
Bolitoglossa morio	Guatemala: Esquintla	USAC 1533	GRF015	KJ787695	
Bolitoglossa morio	Guatemala: Esquintla	USAC 1534	GRF016	KJ787696	
Bolitoglossa morio	Guatemala: Esquintla	USAC 1535	GRF017	KJ787697	
Bolitoglossa morio	Guatemala: Esquintla	USAC 1536	GRF018	KJ787698	
Bolitoglossa morio	Guatemala: Esquintla	USAC 1537	GRF019	KJ787699	
Bolitoglossa morio	Guatemala: Esquintla	USAC 1538	GRF020	KJ787700	
Bolitoglossa morio	Guatemala: Esquintla	USAC 1539	GRF021	KJ787701	
Bolitoglossa morio	Guatemala: Esquintla	USAC 1540	GRF022	KJ787702	
Bolitoglossa morio	Guatemala: Esquintla	USAC 1541	GRF023	KJ787703	
Bolitoglossa morio	Guatemala: Esquintla	USAC 1542	GRF024	KJ787704	
Bolitoglossa morio	Guatemala: Esquintla	USAC 1543	GRF025	KJ787705	
Bolitoglossa morio	Guatemala: Esquintla	USAC 1544	GRF026	KJ787706	
Bolitoglossa pacaya	Guatemala: Esquintla	USAC 1545	GRF027	KJ787707	KJ787751
Bolitoglossa morio	Guatemala: Esquintla	USAC 1546	GRF028	KJ787708	KJ787752
Bolitoglossa morio	Guatemala: Esquintla	USAC 1547	GRF029	KJ787709	
Bolitoglossa morio	Guatemala: Esquintla	USAC 1548	GRF030	KJ787710	
Bolitoglossa morio	Guatemala: Esquintla	USAC 1549	GRF031	KJ787711	
Bolitoglossa morio	Guatemala: Esquintla	USAC 1550	GRF032	KJ787712	
Bolitoglossa morio	Guatemala: Esquintla	USAC 1551	GRF033	KJ787713	
Bolitoglossa morio	Guatemala: Esquintla	USAC 1552	GRF034	KJ787714	
Bolitoglossa morio	Guatemala: Esquintla	USAC 1553	GRF035	KJ787715	
Bolitoglossa morio	Guatemala: Esquintla	USAC 1554	GRF036	KJ787716	
Bolitoglossa morio	Guatemala: Esquintla	USAC 1555	GRF037	KJ787717	
Bolitoglossa morio	Guatemala: Esquintla	USAC 1556	GRF038	KJ787718	
Bolitoglossa morio	Guatemala: Esquintla	USAC 1557	GRF039	KJ787719	
Bolitoglossa morio	Guatemala: Esquintla	USAC 1558	GRF040	KJ787720	
Bolitoglossa morio	Guatemala: Esquintla	USAC 1559	GRF041	KJ787721	
Bolitoglossa morio	Guatemala: Esquintla	USAC 1560	GRF042	KJ787722	
Bolitoglossa morio	Guatemala: Esquintla	USAC 1561	GRF043	KJ787723	
Bolitoglossa pacaya	Guatemala: Esquintla	USAC 1562	GRF044	KJ787724	KJ787753
Bolitoglossa morio	Guatemala: Esquintla	USAC 1563	GRF045	KJ787725	
Bolitoglossa morio	Guatemala: Esquintla	USAC 1564	GRF046	KJ787726	
Bolitoglossa morio	Guatemala: Esquintla	USAC 1565	GRF047	KJ787727	
Bolitoglossa morio	Guatemala: Esquintla	USAC 1566	GRF048	KJ787728	
Bolitoglossa morio	Guatemala: Esquintla	USAC 1568	GRF050	KJ787729	KJ787754
Bolitoglossa morio	Guatemala: Esquintla	USAC 1569	GRF051	KJ787730	
Bolitoglossa morio	Guatemala: Esquintla	USAC 1570	GRF052	KJ787731	
Bolitoglossa morio	Guatemala: Esquintla	USAC 1571	GRF053	KJ787732	

Species	Locality	Voucher #	Field ID	Genebank 16S	Genebank Cyt-b
Bolitoglossa morio	Guatemala: Esquintla	USAC 1573	GRF055	KJ787733	
Bolitoglossa pacaya	Guatemala: Esquintla	USAC 1574	GRF056	KJ787734	
Bolitoglossa pacaya	Guatemala: Esquintla	USAC 1575	GRF057	KJ787735	
Bolitoglossa morio	Guatemala: Esquintla	USAC 1584	GRF066	KJ787736	
Bolitoglossa morio	Guatemala: Esquintla	USAC 1585	GRF067	KJ787737	
Bolitoglossa pacaya	Guatemala: Esquintla	USAC 1586	GRF068	KJ787738	
Bolitoglossa morio	Guatemala: Esquintla	USAC 1587	GRF069	KJ787739	
Bolitoglossa pacaya	Guatemala: Esquintla	USAC 1588	GRF070	KJ787740	KJ787755
Bolitoglossa morio	Guatemala: Esquintla	USAC 1594	GRF075	KJ787741	
Bolitoglossa morio	Guatemala: Esquintla	USAC 1595	GRF076	KJ787742	
Bolitoglossa morio	Guatemala: Esquintla	USAC 1596	GRF077	KJ787743	
Bolitoglossa pacaya	Guatemala: Esquintla	USAC 1597	GRF078	KJ787744	
Bolitoglossa pacaya	Guatemala: Esquintla	USAC 1598	GRF079	KJ787745	
Bolitoglossa pacaya	Guatemala: Esquintla	USAC 1599	GRF080	KJ787746	
Bolitoglossa pacaya	Guatemala: Esquintla	USAC 1600	GRF081	KJ787747	KJ787756
Bolitoglossa morio	Guatemala: Esquintla	USAC 1601	GRF082	KJ787748	
Bolitoglossa morio	Guatemala: Esquintla	USAC 1602	GRF083	KJ787749	
Bolitoglossa morio	Guatemala: Esquintla	USAC 1603	GRF084	KJ787750	
